# Nursing Students' Perceptions of a Clinical Simulation Experience in Community Care: Strengths and Limitations

**DOI:** 10.1002/nop2.70807

**Published:** 2026-09-24

**Authors:** Piedad Gómez‐Torres, Sergio Galarreta‐Aperte, Isabel Blázquez‐Ornat, Isabel Antón‐Solanas, Enrique Ramón‐Arbués

**Affiliations:** ^1^ Department of Nursing Faculty of Health Sciences, University of Granada Ceuta Spain; ^2^ SAPIENF Research Group (B53_23R), University of Zaragoza Zaragoza Spain; ^3^ Department of Physiatry and Nursing Faculty of Health Sciences, University of Zaragoza Zaragoza Spain; ^4^ Department of Physiatry and Nursing Faculty of Health Sciences, San Jorge University Zaragoza Spain

## Abstract

**Aim:**

To explore nursing students' perceptions of and satisfaction with a clinical simulation experience in community care.

**Design and Methods:**

A descriptive mixed‐methods study was conducted at a private university in Spain with 103 s‐year nursing students during the academic year 2023–2024. Four simulation scenarios were designed to represent community care settings. Quantitative data were collected using the Simulation Design Scale, and qualitative data were collected through reflective narratives. Data were analysed using SPSS and ATLAS.ti.

**Results:**

The students reported a high level of satisfaction. The main perceived strengths included the scenarios' close resemblance to real‐world practice, the opportunity to apply theoretical knowledge, the development of clinical reasoning and communication skills, psychological safety and collaborative learning. The main limitations concerned facilitator support, session timing, pre‐simulation preparation, students' anxiety when performing individually in front of others, and limited opportunities for active participation.

**Conclusions:**

Clinical simulation in community care scenarios was perceived as an effective and engaging strategy for developing both technical and non‐technical competencies. Its potential lies in combining realistic and structured scenarios, although aspects such as time management, preparation and instructor support should be strengthened to maximize educational impact.

**Implications for the Profession:**

Our findings reinforce the value of clinical simulation as an educational strategy for preparing future nursing professionals to address the challenges of community‐based practice. Students' positive perceptions of the realism and applicability of the simulation, together with its contribution to the development of clinical, communication and reflective skills, suggest that such experiences can facilitate theory–practice integration and strengthen decision‐making in community care contexts. Furthermore, by identifying areas for improvement, this study provides practical recommendations for optimizing the instructional design of future simulation‐based learning experiences. These enhancements may contribute to improving the quality of nursing education and fostering safer, more empathetic and more competent professional practice aligned with the core principles of primary health care.

**Reporting Method:**

The EQUATOR Network guidelines were followed, specifically the Strengthening the Reporting of Observational Studies in Epidemiology (STROBE) guideline for the quantitative component of the study. Given the mixed‐methods design, reporting also adhered to the Good Reporting of A Mixed Methods Study (GRAMMS) recommendations. There was no patient or public involvement in the design, conduct, reporting, or dissemination of this study.

## 1 Introduction

The increasing complexity of nursing care requires substantial changes in clinical education. Traditional approaches that focus primarily on performing specific technical skills do not adequately develop essential competencies such as clinical reasoning, critical judgement and independent decision‐making (Jeffries and Rizzolo 2006). Innovative educational methods are therefore needed to promote experiential, reflective and student‐centred learning while reproducing the complexity of real clinical environments.

Clinical simulation (CS) has emerged as an important method for integrating theoretical and practical knowledge in a safe environment. It allows students to develop clinical, communication, and decision‐making competencies without compromising patient safety (Kashif and Anny 2024; Shormana 2024). Previous studies have also shown that simulation modules can improve nursing students' achievement, satisfaction and confidence in specific learning contexts, including routine nursing practice (Abd Elfatah et al. 2018, 2019; Hassan et al. 2018). By recreating clinically relevant situations, CS can improve students' preparedness for practice (Sawin et al. 2024). Simulation may involve several modalities, including robotic simulators, which primarily support technical skill development, and standardized patients, who facilitate the development of nontechnical and communication skills (Ward‐Gaines et al. 2021).

### 1.1 Competencies, Phases and Realism in Clinical Simulation

A simulation session typically comprises three phases: prebriefing, during which the objectives and case context are presented, and roles and a safe learning environment are established (Zambrano Guzmán et al. 2019); scenario enactment, during which the main clinical activity occurs; and debriefing, consisting in a guided group reflection in which both participants and observers share perspectives and learn from the experience.

A major advantage of CS is its capacity to develop both technical skills and broader competencies required for clinical practice, including time management, leadership, care prioritization, problem‐solving, teamwork and communication (Gaspar and Banayat 2024). It also turns errors into learning opportunities by helping students identify areas for improvement, receive immediate feedback and strengthen critical thinking, decision‐making and self‐confidence (Kashif and Anny 2024; Shormana 2024).

Simulation fidelity is essential for creating an immersive and meaningful learning experience and depends on a realistic environment, natural interactions and a plausible clinical scenario (Coro‐Montanet et al. 2020). However, sometimes, not all students can participate directly in the same scenario. Students therefore assume one of two roles: participants, who engage directly in the simulation or observers, who follow the case in person or through video transmission (Hungwei and Hill 2020; Schreiber et al. 2020). Although research indicates that participants and observers frequently achieve comparable outcomes in knowledge acquisition, skill development and learning flow, observers should be assigned a structured, active role rather than remain passive viewers (Flo and Solheim 2025; Ham et al. 2024). These different roles may also create tension, particularly for students who perform while being observed by peers and instructors, and who may experience uncertainty or fear of making mistakes (de Dios Duarte et al. 2017). Prebriefing and debriefing can mitigate these emotions by establishing a fiction contract and a psychologically safe environment in which simulation is recognized as a valid representation of clinical practice. This preparation helps students focus on the target competencies and understand that mistakes are treated as learning opportunities rather than grounds for judgement (Muñoz‐Santanach 2022).

### 1.2 Community Care Simulation (CCS)

CS has traditionally focused on hospital settings, such as operating rooms, emergency departments and critical care units, where scenarios typically involve acute conditions and complex procedures. In contrast, community care emphasizes health promotion, disease prevention, long‐term follow‐up and health education, requiring nurses to develop strong communication, planning and decision‐making skills in settings with limited technological resources and considerable human variability. Despite the distinct competencies required in this context, community care remains underrepresented in simulation curricula, limiting students' early exposure to situations commonly encountered at the first level of care (Jefferies and Magnus 2022).

CCS can address preventive and patient‐centred dimensions of care while supporting the application of protocols and health promotion strategies (Raniere De Oliveira Costa et al. 2019). This approach prepares students for the specific demands of a setting in which therapeutic relationships and continuity of care are central. Furthermore, examining how students experience, interpret, and evaluate this methodology can inform the design of future simulation scenarios. Such evidence can strengthen the role of CCS in nursing curricula and help prepare future professionals for contemporary practice (Hung et al. 2021).

### 1.3 The Study

This study aimed to examine nursing students' perceptions of a CCS. Specifically, it assessed students' satisfaction, identified perceived strengths, limitations and opportunities for pedagogical improvement, and explored which aspects they considered meaningful for their future practice.

## 2 Methodology

### 2.1 Study Design, Sample and Setting

This descriptive, cross‐sectional study examined nursing students' perceptions of and satisfaction with a simulation experience set in four CCS scenarios. Of the 162 students enrolled in a Health Education course at a private university in Spain during the 2023–2024 academic year, 103 voluntarily agreed to take part in this investigation (participation rate: 63.6%). This 6‐ECTS course is taught in the second year of the nursing program and develops competencies in communication, health education and health promotion that are particularly relevant to community care.

Four faculty members designed the activity collaboratively. They selected four topics, namely diabetes, hypertension, vaccination schedules and contraceptive counselling, and developed the corresponding scenarios for implementation in the university's high‐fidelity simulation laboratory and debriefing room. Participation was voluntary and did not affect course grades. The activity took place at the end of the semester, after the relevant theoretical content had been taught, and comprised three phases: prebriefing (case presentation and clarification of questions), simulation (scenario enactment) and debriefing (reflective analysis with faculty and students).

The 162 students enrolled in the course were divided into eight groups (A–H), with 20 students in each group except Group G, which comprised 22 students. Each group completed all four scenarios. For each scenario, one student voluntarily assumed the role of a primary care nurse, with active participation allocated according to registration order. Thus, four students from each group participated directly, each in a different scenario, while the remaining students observed. Accordingly, each scenario involved one active participant and 19 observers in groups of 20 students, and one active participant and 21 observers in Group G. All students joined the subsequent debriefing, regardless of their role. Session length varied slightly among groups according to the progression of each scenario, but all sessions remained within the timeframe established by the faculty. Each session lasted approximately 45 min: 5–10 min for prebriefing, 10–20 min for simulation and 20–30 min for debriefing.

### 2.2 Proposed Scenarios

Four scenarios addressed course topics frequently encountered in primary care nursing consultations. Table 1 describes each scenario and the actions expected of students.

**TABLE 1:** Simulation scenarios and objectives.

Learning outcome	Students will be able to systematically apply the nursing process and initiate health education tasks with typical Primary Health Care patients.	
Scenario	Case development	Expected actions
Scenario name: Antonio Context: Nursing consultation. Presentation: a 52‐year‐old hypertensive male, on enalapril 10 mg for the past two years. He missed his last two appointments due to lack of time off work. Actors: ‐ [Lecturer] A 52‐year‐old nurse with extensive experience in Primary Health Care (patient). ‐ Student enrolled in the health education course (nurse).	‐ The patient appears nervous, in a hurry, constantly checking his watch and phone. ‐ He expresses difficulty in changing habits due to work constraints. ‐ He struggles to commit to goals set during the consultation. ‐ First blood pressure reading: 161/86. Second reading: 150/81. ‐ Non‐smoker. ‐ Occasionally plays padel on weekends with friends. ‐ Eats at a nearby restaurant serving ‘homestyle’ food and drinks several coffees a day. ‐ Sometimes forgets to take his medication.	‐ Welcome, introduction and explanation of the purpose of the consultation. ‐ Complete anamnesis: personal and family history, allergies, habits, etc. ‐ Measure blood pressure after waiting a few minutes and repeat the reading. ‐ Explore the patient's knowledge and beliefs regarding hypertension. ‐ Clarify misconceptions. ‐ Assess motivation for behaviour change and provide motivation if necessary. ‐ Basic health education and warning signs. ‐ Establish shared goals. Key points: diet and treatment adherence. ‐ Schedule the next appointment.
Scenario name: Raulito Context: Paediatric nursing consultation. Presentation: A 6‐month‐old infant accompanied by his mother, attending the 6‐month well‐child visit. All previous check‐ups have been completed. The mother brings the child's health booklet with prior data. The infant is currently exclusively breastfed. Actors: ‐ [Lecturer] A 35‐year‐old nurse with extensive experience in Primary Health Care (mother). ‐ Student enrolled in the health education course (paediatric nurse). ‐ High‐fidelity paediatric mannequin (infant).	The mother has many questions: ‐ Anthropometry: she wants to know if the baby's weight is appropriate and is very interested in percentiles, as discussed by other mothers. ‐ Feeding: she wants to know when and how to start complementary feeding, whether she can give solids or purées and whether to give water. ‐ Sleep: the baby still wakes several times at night to breastfeed; she's unsure if this is normal. She co‐sleeps for convenience. ‐ Vaccination: she wants to know which vaccines are due today and whether they might cause a reaction.	‐ Welcome, introduction and explanation of the purpose of the consultation. ‐ Review previously recorded data in the child's health booklet. ‐ Measure height, weight and head circumference, and assess percentiles. ‐ Clarify the mother's questions and misconceptions; provide education on anthropometry, feeding and sleep. ‐ Basic health education: warning signs, accident prevention tips for infancy and psychomotor development (e.g., babywearing, tummy time). ‐ Identify the vaccines required at this check‐up. ‐ Schedule the next visit.
Scenario name: Jose Luis Context: Nursing consultation. Presentation: a 55‐year‐old male recently diagnosed with Type 2 diabetes during a routine occupational health check. He is on valsartan and has just started treatment with metformin. Referred by his family physician for clarification about the condition and lifestyle modifications. Actors: ‐ [Lecturer] A 55‐year‐old nurse with extensive experience in Primary Health Care (patient). ‐ Student enrolled in the health education course (nurse).	‐ The patient is unmotivated. ‐ He has limited knowledge about the disease. ‐ He reports having little time for physical activity. ‐ He is a smoker and notes that job‐related stress causes him to smoke more at times. ‐ He often forgets to take his medication. ‐ He shows little willingness to lose weight.	‐ Welcome, introduction and explanation of the purpose of the consultation. ‐ Complete anamnesis: personal and family history, allergies, habits, etc. ‐ Measure blood pressure, blood glucose, height, weight and waist circumference. ‐ Explore the patient's knowledge and beliefs regarding Type 2 diabetes. ‐ Clarify misconceptions. ‐ Assess motivation for behaviour change and provide motivation if necessary. ‐ Basic health education and warning signs. ‐ Establish shared goals. Key points: diet, smoking cessation, treatment adherence. ‐ Schedule the next appointment.
Scenario name: Maite Context: Midwife consultation. Presentation: A 44‐year‐old woman, mother of three children aged 15, 13 and 2, attending for advice regarding different contraceptive options. Actors: ‐ [Lecturer] A 44‐year‐old midwife with extensive experience in Primary Health Care (patient). ‐ Student enrolled in the health education course (midwife).	‐ Has never used contraceptives. ‐ No lipid disorders. ‐ Not hypertensive. ‐ Normal cervical smear results. ‐ Recently experienced hormonal imbalances (manifested as metrorrhagia and menorrhagia). ‐ Generally, in good health. ‐ Occasionally experiences anaemia, which resolves with Tardyferon. ‐ Has an active sex life, exclusively with her husband.	‐ Welcome, introduction and explanation of the purpose of the consultation. ‐ Complete anamnesis: personal and family history, allergies, habits, etc. ‐ Measure blood pressure, height and weight. ‐ Explore Maite's knowledge and beliefs about contraceptive methods. ‐ Clarify misconceptions. ‐ Explain the different available methods that may be suitable for Maite. ‐ Explore the woman's preferred methods. ‐ Referral to family planning services.^a^ ‐ Arrange a follow‐up appointment.

^a^ In Spain, midwives provide education and counselling on contraception, but they do not prescribe contraceptive methods.

### 2.3 Data Collection

Students' perceptions of and satisfaction with the activity were assessed quantitatively and qualitatively. For the quantitative component, students completed the Simulation Design Scale (SDS), a 20‐item Likert‐type instrument (1 = strongly disagree; 5 = strongly agree). The scale assesses five dimensions of the simulation experience: objectives and information (five items), facilitator support (four items), problem‐solving opportunities (five items), feedback (four items) and fidelity/realism (two items). The instrument has demonstrated strong internal consistency in its original American version (Cronbach's alpha = 0.92 overall) (Jeffries and Rizzolo 2006; Macdonald 2015) and among Spanish nursing students (Cronbach's alpha = 0.95) (Román‐Cereto et al. 2022).

For the qualitative component, students described their perceptions and learning experiences in reflective narratives guided by five questions: (1) How did your participation in the simulation experience contribute to your understanding of nursing practice in community care? (2) Did you encounter any unexpected challenges or obstacles during the activity? (3) What do you consider to be the strengths of this simulation activity? (4) What do you consider to be its weaknesses or areas for improvement? (5) What lessons or insights are you taking away from this experience? The research team developed these questions specifically for the study, in accordance with its educational objectives and its focus on learning through CCS. Informed by general principles of reflective learning and simulation debriefing, the questions explored the contribution of the experience to students' understanding of nursing practice, the difficulties they encountered, the activity's strengths and limitations and their main learning outcomes. Open‐ended wording encouraged personal, critical and in‐depth reflection.

### 2.4 Data Analysis

Quantitative data were summarized using frequencies and percentages or means and standard deviations, as appropriate. Internal consistency was assessed using Cronbach's alpha and was excellent for the overall questionnaire (*α* = 0.977). Analyses were conducted in SPSS version 28.0 (IBM, New York, NY, USA).

The qualitative analysis followed the guidelines of Taylor et al. (2015). Each response was assigned an alphanumeric code and analysed line by line in ATLAS.ti. Systematic coding identified key concepts, themes and emerging patterns, which were refined and organized into categories through iterative reading. Two authors conducted the analysis, and a third researcher resolved any differences in interpretation. The initial reflective questions guided the analysis throughout.

After the quantitative and qualitative data had been analysed separately, the findings were integrated at the interpretation stage using a convergent approach. SDS results were compared with the themes identified in the reflective narratives. Examining convergence and complementarity between the two data sources contextualized the quantitative findings and provided a more comprehensive account of students' perceptions.

### 2.5 Ethical Considerations

All procedures complied with the ethical principles of the Declaration of Helsinki. The Ethics Committee of Universidad San Jorge approved the study (Project ID 89/5/23–24). Before completing the questionnaire, students provided written informed consent and voluntarily authorized the use of their reflective narratives for research purposes. Participation was voluntary and did not affect course grades.

## 3 Results

The 103 participants were predominantly women (80.6%) and had a mean age of 23.4 ± 6.2 years. Only 15 students (14.5%) had no previous simulation experience.

### 3.1 Activity Evaluation

Students reported high levels of agreement with the SDS items, with mean domain scores of approximately 4.5. Support received the lowest score (4.49 ± 0.65), whereas Objectives and Information received the highest (4.54 ± 0.61) (Table 2). Analysis of the reflective narratives identified five themes. Students described the scenarios as realistic and educationally valuable because they supported the application of theoretical knowledge, clinical reasoning, communication practice and collaborative learning in a safe environment. Reported limitations included session length and scheduling, insufficient preparation in some cases, the need for greater facilitator support, anxiety about being observed by peers, and limited opportunities for direct participation.

**TABLE 2:** Students' perceptions of the activity*.

	Simulation Design Scale (SDS)	Strongly disagree	Disagree	Undecided	Agree	Strongly agree	Mean ± SD	Cronbach's Alpha
Objectives and information	1. There was enough information provided at the beginning of the simulation to provide direction and encouragement	1 (1.0%)	1 (1.0%)	9 (8.7%)	23 (22.3%)	69 (67.0%)	4.53 ± 0.77	0.920
	2. I clearly understood the purpose and objectives of the simulation	1 (1.0%)	0 (0.0%)	4 (3.9%)	31 (30.1%)	67 (65.0%)	4.58 ± 0.66	
	3. The simulation provided enough information in a clear matter for me to problem‐solve the situation	1 (1.0%)	0 (0.0%)	6 (5.8%)	34 (33.0%)	62 (60.2%)	4.51 ± 0.69	
	4. There was enough information provided to me during the simulation	1 (1.0%)	0 (0.0%)	6 (5.8%)	29 (28.2%)	67 (65.0%)	4.56 ± 0.69	
	5. The cues were appropriate and geared to promote my understanding	1 (1.0%)	1 (1.0%)	4 (3.9%)	31 (30.1%)	66 (64.1%)	4.55 ± 0.71	
	Average						4.54 ± 0.61	
Support	6. Support was offered in a timely manner	1 (1.0%)	1 (1.0%)	5 (4.9%)	36 (35.0%)	60 (58.3%)	4.48 ± 0.72	0.903
	7. My need for help was recognized	1 (1.0%)	4 (3.9%)	6 (5.8%)	38 (36.9%)	54 (52.4%)	4.35 ± 0.83	
	8. I felt supported by the teacher's assistance during the simulation	1 (1.0%)	1 (1.0%)	4 (3.9%)	34 (33.0%)	63 (61.2%)	4.52 ± 0.71	
	9. I was supported in the learning process	1 (1.0%)	1 (1.0%)	3 (2.9%)	28 (27.2%)	70 (68.0%)	4.60 ± 0.69	
	Average						4.49 ± 0.65	
Problem‐solving	10. Independent problem‐solving was facilitated	1 (1.0%)	2 (1.9%)	4 (3.9%)	31 (30.1%)	65 (63.1%)	4.52 ± 0.75	0.901
	11. I was encouraged to explore all possibilities of the simulation	1 (1.0%)	1 (1.0%)	5 (4.9%)	33 (32.0%)	63 (61.2%)	4.51 ± 0.72	
	12. The simulation was designed for my specific level of knowledge and skills	1 (1.0%)	1 (1.0%)	8 (7.8%)	37 (35.9%)	56 (54.4%)	4.41 ± 0.76	
	13. The simulation allowed me the opportunity to prioritize nursing assessments and care	1 (1.0%)	0 (0.0%)	4 (3.9%)	29 (28.2%)	69 (67.0%)	4.60 ± 0.66	
	14. The simulation provided me an opportunity to goal set for my patient	1 (1.0%)	0 (0.0%)	4 (3.9%)	36 (35.0%)	62 (60.2%)	4.53 ± 0.66	
	Average						4.52 ± 0.60	
Feedback/Guided Reflection	15. Feedback provided was constructive	1 (1.0%)	1 (1.0%)	5 (4.9%)	28 (27.2%)	68 (66.0%)	4.56 ± 0.72	0.930
	16. Feedback was provided in a timely manner	1 (1.0%)	0 (0.0%)	6 (5.8%)	31 (30.1%)	65 (63.1%)	4.54 ± 0.69	
	17. The simulation allowed me to analyse my own behaviour and actions	1 (1.0%)	0 (0.0%)	3 (2.9%)	41 (39.8%)	58 (56.3%)	4.50 ± 0.65	
	18. There was an opportunity after the simulation to obtain guidance/feedback from the teacher to build knowledge to another level	1 (1.0%)	0 (0.0%)	6 (5.8%)	32 (31.1%)	64 (62.1%)	4.53 ± 0.69	
	Average						4.54 ± 0.63	
Fidelity (realism)	19. The scenario resembled a real‐life situation	1 (1.0%)	2 (1.9%)	2 (1.9%)	30 (29.1%)	68 (66.0%)	4.57 ± 0.72	0.935
	20. Real life factors, situations and variables were built into the simulation scenario	1 (1.0%)	0 (0.0%)	4 (3.9%)	30 (29.1%)	68 (66.0%)	4.59 ± 0.66	
	Average						4.58 ± 0.67	
SDS average							4.53 ± 0.59	0.977

* Students: *n* = 103.

The five themes captured both strengths and areas for improvement in the activity's design and implementation:

(a) Strength: Realism and practical application of theory.

Participants perceived the clinical cases as authentic and representative of situations commonly encountered in practice. This alignment with real‐world practice enabled them to apply theoretical knowledge in context, bridging the gap between theory and practice and reinforcing their learning.

You prepare for similar scenarios that you'll have to face in day‐to‐day professional practice.

It's a good way to learn because you apply the theory learned in class, which helps consolidate the knowledge.

(b) Strength: Development of clinical reasoning and communication skills.

Students viewed the experience as an opportunity to exercise clinical judgement and strengthen patient communication. They particularly valued situations requiring empathy, decision‐making and health promotion.

It's not as easy as it seems to care for a patient, give advice and education… how to follow up, how to care for them and above all the empathy involved in how you speak to them.

The most positive aspect was definitely learning how to approach different cases with empathy and by encouraging patient self‐care.

(c) Strength and limitation: Emotionally safe environment and anxiety during exposure.

Students particularly valued the opportunity to practice in a safe setting without fear of making mistakes, which strengthened their self‐confidence. One student highlighted: ‘Being able to put yourself in the situation without being afraid of messing up’.

At the same time, performing alone in front of the group created discomfort, vulnerability and nervousness for some students: ‘The worst part was going up alone; it's more embarrassing’. ‘Some of us feel embarrassed when doing the simulation’.

(d) Limitation: Organization, timing and prior preparation.

Students identified several organizational issues, including the length of some cases, the concentration of multiple activities on one day, and insufficient preparation. They associated these conditions with fatigue, poorer performance, and uncertainty during the activity. One student observed: ‘It's too many hours in a row; it could have been split into two sessions’. Another noted: ‘In some cases, we hadn't covered the theory yet or didn't know what to do, so when we entered the simulation space, we felt a bit lost’. Some also felt that lengthy cases disrupted the activity's pace: ‘Very long cases, and that made it a bit tiring’.

(e) Strength and limitation: Collaborative learning and unequal active participation.

Students described the CCS experience as promoting teamwork and peer exchange, which enriched their learning. One student valued the opportunity of ‘…helping each other as classmates and creating that team dynamic’, while another valued ‘…sharing different points of view with classmates’.

However, some students were dissatisfied with the limited opportunities for direct participation. As one explained: ‘I would like there to be more opportunities to participate, since with only four cases, not everyone gets the opportunity to participate’.

## 4 Discussion

Studies using the Simulation Design Scale (SDS) have consistently reported positive student evaluations of clinical simulation. In the present study, the scale demonstrated excellent internal consistency (Cronbach's *α* = 0.98), supporting the reliability of scores across its dimensions. Consistent with our findings, nursing and medical students in Brazil reported high scores (≥ 4.60) in all dimensions, particularly fidelity/realism and problem‐solving (De Oliveira Costa et al. 2019; Pereira Quadros et al. 2021). Similar patterns have been observed in other geographic settings, where mean ratings exceeded 4 in every dimension (Ma et al. 2024; Olaussen et al. 2020).

The findings identified clear strengths and limitations of the CCS experience. Students valued the scenarios' fidelity and clinical relevance, the integration of theory and practice, the development of communication and clinical reasoning skills and the opportunity to learn collaboratively in a safe environment. The principal limitations were organizational and pedagogical, namely lengthy and concentrated sessions, insufficient preparation for some scenarios, anxiety about performing in front of peers, limited opportunities for direct participation and a need for more individualized facilitator support. The perceived educational value of simulation therefore appeared to depend not only on scenario fidelity but also on careful planning, structured prebriefing, equitable participation and tailored feedback.

Twenty‐one students reported that the number of cases per session and the participation of only one student in each case restricted their involvement. Prebriefing should therefore emphasize that both direct participation and observation can support meaningful learning (Persico et al. 2025). When paired with structured debriefing, active observation can itself provide a valuable educational experience. Explaining this role from the outset may sustain observers' motivation and encourage their engagement in subsequent analysis (Hungwei and Hill 2020; Schreiber et al. 2020). Such engagement can also strengthen collaborative learning in simulated environments (Rogers et al. 2020).

Although students generally considered the sessions relevant to their learning needs, some perceived a mismatch between scenario demands and their level of knowledge. Two explanations are possible. Students with strong theoretical knowledge but limited practical experience may underestimate their abilities (Karnick et al. 2021); alternatively, scenarios may be overly complex or contain irrelevant details that create cognitive noise. Learning objectives and scenario complexity should therefore be aligned with students' level of preparation, and scenarios should remain clear and clinically credible (Salameh et al. 2021).

Support was the lowest‐rated SDS domain, suggesting that challenging situations created uncertainty and that some students needed more individualized guidance. This finding is consistent with evidence emphasizing close pedagogical interaction (Foronda et al. 2020; Levett‐Jones and Lapkin 2014). Further research could examine common characteristics among students who report lower levels of support and use them to tailor teaching strategies. A psychologically safe environment may also promote participation and help students learn from mistakes (Elendu et al. 2024).

Fidelity/realism received the highest domain rating, consistent with narratives emphasizing the credibility of the cases and fulfilment of the fiction contract. Accurate, naturalistic scenario design has been associated with immersion and perceived learning effectiveness. Simulation also allows educators to observe performance in real time, identify areas for improvement and adapt scenarios to learners' needs as part of continuous quality improvement (Cho et al. 2023; Trisnaningtyas et al. 2024). High ratings for informational clarity and the integration of realistic clinical elements further support this adaptive capacity and are consistent with previous findings (Bergamasco et al. 2018). The simulation team involved in this study maintains active clinical practice, which contributes to the development of authentic and clinically relevant scenarios. When simulation instructors have limited current clinical experience, involving a practicing nurse as a subject‐matter expert may help ensure the clinical accuracy and relevance of the scenarios.

Students perceived CCS as a valuable means of integrating theory and practice while developing technical, communication and decision‐making skills. Simulation may also strengthen self‐confidence and support the transfer of learning to clinical settings (Kashif and Anny 2024; Shormana 2024). It can therefore help prepare confident and competent professionals (Gandhi et al. 2021), while posing no risk to patients (Padilha et al. 2019). A safe and confidential environment may encourage students to participate without fear of error or judgement, as reflected in themes (a), (b), (c) and (e) of the narrative analysis. Previous studies, likewise, have found that simulation modules improved nursing students' achievement and satisfaction, although those studies focused on maternity or normal labor rather than community care (Abd‐Elfattah et al. 2018; Abd Elfatah et al. 2019; Hassan et al. 2018).

The prebriefing promoted interaction, collaboration and the exchange of experiential knowledge; competencies that support the multidisciplinary nature of community care. Nevertheless, performing individually while being observed and evaluated created anxiety and vulnerability for some students and may have affected their performance (Labrague et al. 2019; Penman et al. 2021). This finding is consistent with evidence that clear scenarios, effective communication and appropriate support facilitate the transfer of learning (Der Sahakian et al. 2024).

Students also criticized the length of some cases and the scheduling of all sessions on a single day, which they associated with fatigue and poorer performance. Heavy workloads can increase anxiety and reduce learning effectiveness (Cox et al. 2016). Distributing sessions across several time points may provide greater opportunity for reflection and focused feedback (McGraw et al. 2019). Future activities should therefore consider spreading scenarios across multiple sessions, providing preparatory materials in advance, assigning structured observer roles, and increasing opportunities for direct participation. These changes may reduce fatigue and anxiety while strengthening engagement and perceived support.

### 4.1 Implications for Nursing Education and Practice

These findings have practical implications for the design and implementation of simulation‐based learning in undergraduate nursing education, particularly within community care settings. Educators should use structured prebriefing to define learning objectives, roles and expectations before each scenario, thereby promoting psychological safety and meaningful engagement (Muñoz‐Santanach 2022; Persico et al. 2025). Facilitators should provide consistent support throughout the activity and tailor their guidance to students' needs because effective facilitation and debriefing are central to high‐quality simulation‐based education (Foronda et al. 2020; Levett‐Jones and Lapkin 2014). Structured observation tasks may increase the educational value of the observer role and encourage active contributions during debriefing; when appropriately guided, observational learning can be as meaningful as direct participation (Ham et al. 2024; Rogers et al. 2020). Future programs should also provide more equitable opportunities for direct participation and ensure that students have sufficient theoretical preparation. Together, these measures may improve students' learning experience and prepare future nurses for the interpersonal, educational and decision‐making demands of community care.

### 4.2 Limitations

Several limitations should be considered. First, the study was conducted at a single private university in Spain, which may limit transferability to other educational settings. Second, voluntary participation may have introduced self‐selection bias because students with more favourable attitudes toward simulation may have been more likely to participate. Third, the study used self‐reported perceptions, satisfaction ratings and reflective narratives but did not objectively assess competence, clinical performance, or learning outcomes. The findings therefore reflect perceived educational value rather than educational effectiveness. Most participants had previous simulation experience, which may also have shaped their perceptions and limited the applicability of the findings to novice learners. In addition, only one student participated directly in each scenario while the others observed, creating unequal opportunities for hands‐on involvement. Finally, although all sessions followed the same educational structure, their duration varied slightly according to scenario progression and may have influenced students' experiences. Future multicenter studies should incorporate objective learning outcomes and simulation formats that provide more equitable opportunities for direct participation.

Overall, students perceived CCS as a valuable educational strategy, particularly for developing therapeutic communication, clinical decision‐making and interpersonal skills. The mixed‐methods approach demonstrated high levels of student satisfaction while providing deeper insight into their experiences and perceptions. However, the findings also highlight opportunities to strengthen organizational and instructional support and to ensure more equitable opportunities for active participation. Despite its potential to prepare students for the complexities of frontline care, CCS remains underrepresented in nursing curricula. Its systematic integration, supported by pedagogical approaches responsive to students' learning needs, may enhance nursing education and better prepare future nurses to deliver high‐quality community care.

## 5 Conclusions

Nursing students perceived CCS as a valuable educational strategy. Its principal strengths were the fidelity and clinical relevance of the scenarios, theory–practice integration, collaborative learning, communication skill development and the opportunity to practice the professional role in a safe environment. The main limitations concerned instructional support, time management, prior preparation, anxiety when performing in front of others and unequal opportunities for direct participation. Addressing these limitations may improve the experience and increase its educational impact.

Community care requires professionals with strong interpersonal skills, autonomy and adaptability; simulation scenarios that reflect this complexity should therefore be systematically incorporated into nursing education. These findings can inform training approaches that respond more closely to students' needs and better prepare them for the challenges of community‐based care.

## Author Contributions

**Piedad Gómez‐Torres:** writing – original draft, writing – review and editing, data curation. **Sergio Galarreta‐Aperte:** writing – original draft, writing – review and editing, data curation. **Isabel Blázquez‐Ornat:** writing – original draft, writing – review and editing, data curation. **Isabel Antón‐Solanas:** writing – review and editing. **Enrique Ramón‐Arbués:** conceptualization, investigation, methodology.

## Funding

The authors have nothing to report.

## Disclosure

The authors affirm that the methods used in the data analyses are suitably applied to their data within their study design and context, and the statistical findings have been implemented and interpreted correctly. The authors agree to take responsibility for ensuring that the choice of statistical approach is appropriate and is conducted and interpreted correctly as a condition to submit to the Journal.

## Conflicts of Interest

The authors declare no conflicts of interest.

## Data Availability

The data that support the findings of this study are available from the corresponding author upon reasonable request.
